# Congenital Chagas Disease in the United States: The Effect of Commercially Priced Benznidazole on Costs and Benefits of Maternal Screening

**DOI:** 10.4269/ajtmh.20-0005

**Published:** 2020-02-24

**Authors:** Victoria Perez-Zetune, Stephanie R. Bialek, Susan P. Montgomery, Eileen Stillwaggon

**Affiliations:** 1 Department of Economics, University of Maryland, College Park, Maryland;; 2 Parasitic Diseases Branch, Division of Parasitic Diseases and Malaria, Center for Global Health, Centers for Disease Control and Prevention, Atlanta, Georgia;; 3 Department of Economics, Gettysburg College, Gettysburg, Pennsylvania

## Abstract

Chagas disease, caused by *Trypanosoma cruzi*, is transmitted by insect vectors, and through transfusions, transplants, insect feces in food, and mother to child during gestation. An estimated 30% of infected persons will develop lifelong, potentially fatal cardiac or digestive complications. Treatment of infants with benznidazole is highly efficacious in eliminating infection. This work evaluates the costs of maternal screening and infant testing and treatment for Chagas disease in the United States, including the cost of commercially available benznidazole. We compare costs of testing and treatment for mothers and infants with the lifetime societal costs without testing and consequent morbidity and mortality due to lack of treatment or late treatment. We constructed a decision-analytic model, using one tree that shows the combined costs for every possible mother–child pairing. Savings per birth in a targeted screening program are $1,314, and with universal screening, $105 per birth. At current screening costs, universal screening results in $420 million in lifetime savings per birth-year cohort. We found that a congenital Chagas screening program in the United States is cost saving for all rates of congenital transmission greater than 0.001% and all levels of maternal prevalence greater than 0.06% compared with no screening program.

Chagas disease, caused by the protozoan parasite *Trypanosoma cruzi*, can cause serious, even fatal, cardiac and gastrointestinal damage in 30% of persons infected. Maternal transmission is becoming an increasingly important share of new infections as the success of other control measures continues. Benznidazole is highly effective in treating infants and effective in treating adolescents and young adults. Previously, we demonstrated that a universal program of screening and treating infants and mothers would be cost saving. Benznidazole is now approved by the Food and Drug Administration (FDA) and is commercially available. We demonstrate that a universal screening and treatment program is still cost saving in the United States, even with commercially priced benznidazole.

In August 2017, the US FDA approved benznidazole, one of two drugs used to treat Chagas disease, for treatment of children ages 2–12 years and by physician discretion outside that age range.^1^ Benznidazole became commercially available on May 14, 2018.^2^ In the June 2018 issue (Vol. 98, No. 6) of this journal (available online April 30, 2018),^3^ we published an economic decision-analytic model evaluating the societal cost of universal screening of pregnant women for Chagas disease in the United States. This report incorporates the commercial cost of treatment with benznidazole into our model of the economic costs and benefits of maternal screening and treatment for mother–child pairs.

Chagas disease is caused by *T. cruzi* transmitted by triatomine insects found in Central and South America, Mexico, and the southern United States.^4^ An estimated 5.7 million people in Latin America are infected with *T. cruzi*, with an additional 400,000 Latin Americans abroad, primarily in the United States (about 300,000), Spain, and Japan.^5–7^ Chagas disease is also spread by blood transfusions, organ transplants, consumption of insect feces in food, and congenital transmission. Because of success in vector control in endemic areas and blood screening, congenital transmission accounts for an increasing share (1/4) of new cases, with approximately 9,000 congenital infections per year in Latin America and several hundred in the United States and Europe.^4,5,8^ An estimated 30% of infected persons will develop lifelong, potentially fatal cardiac or digestive complications.^9,10^

Efficacy of benznidazole treatment for infants younger than 1year is 90–100%, and for adults, efficacy ranges from 40% to 70% depending on age and length of time since infection.^8,11^ Moreover, the risk of congenital transmission is considerably lower for women treated before pregnancy.^12,13^ For infected persons with chronic conditions, treatment has not been found to remediate existing cardiac or digestive tract damage.^14^ A 2018 retrospective study, however, found substantial benefits for persons with chronic Chagas disease who were treated, primarily before the age of 40 years. Compared with persons never treated with benznidazole, the treatment group had an odds ratio of 0.21 for mortality within 2 years, of 0.23 for electrocardiogram (ECG) abnormalities, and of 0.27 for *T. cruzi* DNA on polymerase chain reaction (PCR).^15^

The CDC provided benznidazole treatment at no cost for 369 patients from 2011 to 2018 in 41 states and the District of Columbia.^2^ Patient enrollment in the CDC program ended in May 2018 when Exeltis USA, Inc. (Florham Park, NJ) began commercial supply of benznidazole. Exeltis is a subsidiary of Insud Pharma, which runs a nonprofit foundation, Mundo Sano. Between May 2018 and January 2019, 85 patients with suspected *T. cruzi* infection were treated in 19 states (Exeltis).

To date, there is no maternal screening policy for Chagas disease in the United States. Food and Drug Administration approval of benznidazole for treatment of infants is pending (Exeltis). Early diagnosis could prevent severe and even deadly complications associated with the chronic stage of the disease in infants and their mothers. Pregnant women are the best access point for diagnosing and treating entire families because delivery is the most likely time for contact with the healthcare system. In 2017, Hispanics had the highest uninsured rate, 16%, and the lowest rate of private insurance coverage, 53.5%,^16,17^ and 30% of Hispanic adults reported having no usual source of health care, the highest for any group.^18^ Hispanic women, on the other hand, have the lowest rate of out-of-hospital births of any group in the United States, 0.05%.^19^

Using a societal perspective, we compare the costs of maternal screening and infant testing and treatment as needed (the screening option) with the costs of no screening, with its consequent morbidity and mortality due to lack of treatment or late treatment. We constructed a decision-analytic model for the United States using TreeAge software (TreeAge Software Inc., Williamstown, MA) to find the lower cost option. As before, we use one tree that comprises a decision node (screening or no screening), chance nodes (probabilities of maternal infection, transmission, and various degrees of injury), and terminal nodes that correspond to each possible mother–child outcome. Each outcome represents the expected value of all economic costs based on a series of conditional probabilities for each branch.^3^

Table 1 lists some key parameters for the model. Point estimates and ranges are derived from the literature indicated in the source column. Full description of methods and data sources appears in the original article and its online supplement.^3^ Although there have been new studies of Chagas disease since 2018, none presents new data that would change the model parameters, except the cost of benznidazole.

**Table 1:** Key parameters

Parameter	Name in tree	Point estimate (range)	Sources
Prevalence	Prevalence	0.0131 (0.0–0.0131)	Ref. 5 (ref. 29,30,32)
Mother-to-child transmission rate	MTCT	0.05 (0.0–0.05)	Ref. 6,33,34 (ref. 28,32,35–37)
Benznidazole, infant	Rx_baby	$180	Ref. 1,24
Benznidazole, mother	Rx_mom	$720	Ref. 1,24
Testing cost, infant	Dx_baby	$400	Ref. 3
Testing cost, mother	Dx_mom	$60 ($8–$60)	Table 2, multiple sources in ref. 3 (ref. 20)

The point estimate for screening cost is $60 per pregnancy, but the model also calculates for screening cost as low as $8 per pregnancy through sensitivity analysis. A new point-of-care diagnostic test was approved by the FDA in December 2016 for which the expected price will be $4.00 ($8 with confirmatory test).^20,21^ We list a higher cost for infant PCR than is in effect to maintain the conservative assumptions of the earlier article. Currently, Chagas disease PCR testing is only available at the CDC at no charge. Newborns should be tested at birth and 4–6 weeks of age.^22^ To ensure that no infection is missed by PCR testing, infants who were PCR negative at both intervals should have serologic testing for antibody to *T. cruzi* at the age of 9–10 months, after maternal antibody is no longer present.^22^ The cost saving from a maternal screening program could be especially sensitive to two clinical variables: prevalence among pregnant women and mother-to-child transmission risk. For both prevalence and maternal transmission, we set zero as the lower bound (Table 1).

Cost-to-charge ratios vary in the United States by state, by provider, and by coverage. Exeltis provides benznidazole at a variety of prices (charges), depending on whether the patient has no insurance, inadequate insurance, or Medicare/Medicaid coverage. A collaboration of Insud Pharma, Drugs for Neglected Diseases *initiative*, and Mundo Sano arranged the subsidization of pricing.^23^ Mundo Sano covers the gap so that no patient faces exorbitant costs. For benznidazole, the average Medicare patient full cost is $401, but patient out-of-pocket costs are $34.^24^ Because pharmaceutical pricing in the United States is essentially unrelated to economic cost, we do not use Exeltis charges, but rather the Wholesale Acquisition Cost (WAC). For a full 60-day course of treatment for an infant, the cost is $180, and for the mother, $720.^1,24^

We found that commercial provision of benznidazole made little difference in the cost savings derived from maternal screening for Chagas disease. The savings per birth in a targeted screening program, using prevalence of 0.0131 among Hispanic women from endemic areas, is $1,314, only $10 less than with free benznidazole. We found that a program of universal screening would save $105 per birth, compared with $106 per birth with free benznidazole.^3^ The lower bound maternal prevalence of Chagas disease for screening to be cost saving is 0.06%. The estimated prevalence of Chagas in US women of childbearing age is 0.16%. The results are robust to variations in the rate of mother-to-child transmission down to 0.001%. Table 2 displays these results.

**Table 2:** Results: lifetime societal costs and savings, no screening, and screening

Scenario	$60 screening cost per birth		$8 screening cost per birth	
	Per birth	Per birth-year cohort	Per birth	Per birth-year cohort
Targeted screening, 480,000 births, maternal prevalence of 1.31%				
No screening	$2,321	$1,114,080,000	$2,321	$1,114,080,000
Screening	$1,007	$483,360,000	$955	$453,600,000
Savings	$1,314	$630,720,000	$1,366	$660,480,000
Universal screening, 4,000,000 births, maternal prevalence of 0.16%				
No screening	$279	$1,116,000,000	$279	$1,116,000,000
Screening	$174	$696,000,000	$121	$484,000,000
Savings	$105	$420,000,000	$158	$632,000,000

For screening with two point-of-care tests at $8, the savings per birth is $1,366 in a targeted screening program and $158 per birth with universal screening. With the $8 screening cost, the threshold for screening as the cost-saving option is maternal prevalence of 0.0000757 (0.008%). It is a construct of the decision-tree method that the per-birth savings differ substantially between targeted and universal screening. That is because the per-birth savings are multiplied in each option by the number of births, and the total savings are enormous even in a universal program (Table 2).

Both targeted screening and universal screening are cost saving over a wide range of prevalence and screening costs in the United States. The only parameter that makes an important difference in the amount of savings from the screening option is maternal prevalence. Varying mother-to-child transmission from 0.0 to 0.05 has a trivial effect on the estimate of savings. Thus, even if maternal transmission is extremely low in the United States because of environmental or biological factors, that makes little difference in the large savings from screening. Including the cost of a second infant PCR makes a trivial difference in the result.

Implementation of universal screening with a point-of-care test would cost $70.6 million, including screening cost, benznidazole treatment for mothers and babies, and costs of enhanced care for women with digestive conditions or indeterminate status who would not have been identified in the no-screening scenario. The benefit ($632 million, the discounted present value of the savings) is almost nine times the cost of the screening program for each birth-year cohort (Table 3).

**Table 3:** Lifetime savings per birth cohort, implementation costs (screening, enhanced care, and benznidazole treatment), and benefit–cost ratios

	Targeted (480,000 births)		Universal (4 million births)	
	At $60 per screen	At $8 per screen	At $60 per screen	At $8 per screen
Lifetime savings per birth cohort	$630,270,000	$660,480,000	$420,000,000	$632,000,000
Screening cost	$31,320,000	$6,360,000	$202,520,000	$34,520,000
Enhanced care under screening	$31,528,000	$31,528,000	$31,528,000	$31,528,000
Cost of benznidazole	$4,592,700	$4,592,700	$4,592,700	$4,592,700
Total implementation costs	$67,440,700	$42,480,700	$278,640,700	$70,640,700
Benefit–cost ratio	9.35	15.5	1.5	8.9

Targeted screening toward first- and second-generation Latinas from endemic areas could be less costly, but surveys of obstetricians indicate a lack of information about Chagas disease.^22,25^ Because the Hispanic population is distributed across the United States,^26^ incorporating *T. cruzi* into routine screening may be more feasible. The US political climate makes it risky for many Hispanics to seek medical attention;^27^ universal screening avoids profiling and is likely to be more acceptable to vulnerable women. Pregnant women and newborns are already screened for a wide variety of genetic conditions and congenitally transmitted diseases, including syphilis, HIV, and, in some states, toxoplasmosis, rubella, and cytomegalovirus.^28–30^ Our model demonstrates that, even at current testing costs, maternal screening and follow-up infant testing and treatment as needed are cost saving for maternal prevalence as low as 0.057% and for mother-to-child transmission probability as low as 0.001%. With the new point-of-care test, universal screening is cost saving for prevalence as low as 0.008% of pregnant women. Lifetime societal costs, including direct medical costs and productivity loss due to morbidity and premature mortality, are 8.9 times the cost of implementing a universal maternal screening program. This analysis supports adding testing for *T. cruzi* infection to routine screening in pregnancy or at the time of delivery.

The analysis could have limited application in other countries if subsidized pricing of benznidazole was not available. On the other hand, US pharmaceutical prices are the highest in the world.^31^ A second limitation is the high cost attributed to infant PCR, but a more realistic cost (currently zero) would only increase the estimated savings.

Screening and preventive programs in general are often assumed to be very expensive, especially for low-prevalence conditions. As this study demonstrates, the lifetime costs of morbidity and mortality for infected mothers and babies far outweigh the implementation costs. Moreover, positive serology indicates the need to test the mother’s older children, as well as the newborn, increasing the benefits. After maternal treatment, subsequent pregnancies would be protected from transmission.^12,13^ Given the outstanding benefits in the earlier study,^3^ it is not surprising that the benefits remain even with commercially available benznidazole. Moreover, the drug provider has guaranteed affordable pricing, and relatively few mothers and babies need to be treated. Screening cost, even at present, is low, and it is likely to be much lower in the near future. Logistically, universal screening is preferable to targeted screening and is still cost saving.
